# Frequent loss of heterozygosity at the DNA mismatch-repair loci *hMLH1* and *hMSH3* in sporadic breast cancer

**DOI:** 10.1038/sj.bjc.6690162

**Published:** 1999-03

**Authors:** N Benachenhou, S Guiral, I Gorska-Flipot, D Labuda, D Sinnett

**Affiliations:** Division of Hemato-Oncology, Charles Bruneau Cancer Center, Ste-Justine Hospital, Montreal, Québec, Canada; Division of Pathology, Montreal Hôtel-Dieu Hospital, Montreal, Québec, Canada; Department of Pediatrics, University of Montreal, Montreal, Québec, Canada

**Keywords:** mismatch-repair, *hMLH1*, *hMSH3*, loss of heterozygosity, breast cancer

## Abstract

To study the involvement of DNA mismatch-repair genes in sporadic breast cancer, matched normal and tumoral DNA samples of 22 patients were analysed for genetic instability and loss of heterozygosity (LOH) with 42 microsatellites at or linked to *hMLH1* (3p21), *hMSH2* (2p16), *hMSH3* (5q11–q13), *hMSH6* (2p16), *hPMS1* (2q32) and *hPMS2* (7p22) loci. Chromosomal regions 3p21 and 5q11–q13 were found hemizygously deleted in 46% and 23% of patients respectively. Half of the patients deleted at *hMLH1* were also deleted at *hMSH3*. The shortest regions of overlapping (SRO) deletions were delimited by markers *D3S1298* and *D3S1266* at 3p21 and by *D5S647* and *D5S418* at 5q11–q13. Currently, the genes *hMLH1* (3p21) and *hMSH3* (5q11–q13) are the only known candidates located within these regions. The consequence of these allelic losses is still unclear because none of the breast cancers examined displayed microsatellite instability, a hallmark of mismatch-repair defect during replication error correction. We suggest that *hMLH1* and *hMSH3* could be involved in breast tumorigenesis through cellular functions other than replication error correction. © 1999 Cancer Research Campaign


*British Journal of Cancer* (1999) **79**(7/8), 1012–1017
© 1999 Cancer Research Campaign
Article no. bjoc.1998.0162

### Frequent loss of heterozygosity at the DNA mismatch-repair loci *hMLH1* and *hMSH3* in sporadic breast cancer

**N Benachenhou[1], S Guiral[1], I Gorska-Flipot[2], D Labuda[1,3] and D Sinnett[1,3]**

[1]Division of Hemato-Oncology, Charles Bruneau Cancer Center, Research Center, Ste-Justine Hospital, Montreal, Québec; [2]Division of Pathology, Montreal Hôtel-Dieu Hospital, Montreal, Québec; [3]Department of Pediatrics, University of Montreal, Montreal, Québec, Canada

**Summary** To study the involvement of DNA mismatch-repair genes in sporadic breast cancer, matched normal and tumoral DNA samples of 22 patients were analysed for genetic instability and loss of heterozygosity (LOH) with 42 microsatellites at or linked to *hMLH1* (3p21), *hMSH2* (2p16), *hMSH3* (5q11–q13), *hMSH6* (2p16), *hPMS1* (2q32) and *hPMS2* (7p22) loci. Chromosomal regions 3p21 and 5q11–q13 were found hemizygously deleted in 46% and 23% of patients respectively. Half of the patients deleted at *hMLH1* were also deleted at *hMSH3*. The shortest regions of overlapping (SRO) deletions were delimited by markers *D3S1298* and *D3S1266* at 3p21 and by *D5S647* and *D5S418* at 5q11–q13. Currently, the genes *hMLH1* (3p21) and *hMSH3* (5q11–q13) are the only known candidates located within these regions. The consequence of these allelic losses is still unclear because none of the breast cancers examined displayed microsatellite instability, a hallmark of mismatch-repair defect during replication error correction. We suggest that *hMLH1* and *hMSH3* could be involved in breast tumorigenesis through cellular functions other than replication error correction.

**Keywords:** mismatch-repair; *hMLH1; hMSH3*; loss of heterozygosity; breast cancer

Breast cancer is the most frequent neoplasia that affects women in the Western world. It is a heterogeneous disease, which displays a broad spectrum of clinical and pathological characteristics, and like most solid tumours is thought to develop through the accumulation of genetic alterations leading to uncontrolled cellular growth. Loss of heterozygosity (LOH) studies in non-hereditary breast tumours have shown deletions at a frequency ranging from 20% to 50% in several chromosomal arms (reviewed in Sato et al, 1990; Cornelisse et al, 1992; Bièche and Lidereau, 1995), suggesting the involvement of several tumour-suppressor genes in breast carcinogenesis.

Recently, another type of gene, encoding components of the DNA mismatch-repair system, has been linked to hereditary non-polyposis colorectal cancer (HNPCC) (Fishel et al, 1993; Leach et al, 1993; Bronner et al, 1994; Nicolaides et al, 1994; Papadopoulos et al, 1994, 1995). These genes have been found mutated in HNPCC and are presumably involved in certain sporadic forms of cancer (Leach et al, 1993; Nicolaides et al, 1994; Papadopoulos et al, 1994, 1995; Liu et al, 1995; Risinger et al, 1996). Their defects generally lead to a genome-wide instability of microsatellites in tumoral cells referred to as the replication error (RER) phenotype. In addition to HNPCC, the RER phenotype was observed in a number of sporadic cancers (Han et al, 1993; Speicher, 1995) including breast cancer (Yee et al, 1994; Karnik et al, 1995; Paulson et al, 1996), thus suggesting that deficiency in DNA repair could be involved in breast carcinogenesis. Like suppressor genes (Knudson, 1971), mismatch-repair mutants are inherited as recessive traits that eventually become dominant because of somatic mutations inactivating the second allele. This second mutational step may be revealed as LOH which was reported at the *hMLH1* locus in HNPCC patients (Hemminki et al, 1994) as well as in sporadic colorectal cancers (Tomlinson et al, 1996).

Based on these observations, we examined the involvement of mismatch-repair genes in sporadic breast cancer by microsatellite instability and LOH analyses. We have screened 22 primary breast carcinomas using 42 polymorphic microsatellites within or closely linked to *hMLH1*, *hMSH2*, *hMSH3*, *hMSH6*, *hPMS1* and *hPMS2* loci. We found that *hMLH1* and *hMSH3* were frequently deleted in tumoral cells, suggesting their possible involvement in sporadic breast cancer.

*Received 28 January 1998*
*Revised 21 July 1998*
*Accepted 23 July 1998*

*Correspondence to:* D Sinnett, Centre de Cancérologie Charles Bruneau, Research Center, Sainte-Justine Hospital, 3175 Côte Ste-Catherine, Montréal, Québec, H3T 1C5, Canada

### MATERIALS AND METHODS

### DNA samples

Matched tumoral and normal sample pairs were obtained from 22 breast carcinoma patients (ages 40–90; mean, 58.09; median, 60), including ten metastatic cases, who underwent surgery at the Montreal Hôtel-Dieu Hospital. This is an unselected group of apparent sporadic cases with limited clinical information. Because family histories were unavailable, it was expected that, if any, only 5–10% of the samples would be from patients with a familial form of the disease (Newman et al, 1988). DNA was isolated from fresh material by a standard procedure using digestion with proteinase K and phenol/chloroform extractions.

### Microsatellite analysis

Matched DNA sample pairs were genotyped by polymerase chain reaction (PCR) at the 42 following highly polymorphic (62–90% heterozygosity) microsatellite loci: on chromosome 3p14–p26 (*hMLH1*), *D3S1286*, *D3S1266*, *D3S1745*, *D3S1561*, *D3S1611*, *D3S1612*, *D3S1298*, *D3S1260*, *D3S3559*, *D3S3582*, *D3S3647*, *D3S1588*, *D3S1582*, *D3S1613*, *D3S1234*, *D3S1300* and *D3S1312*; on 5p14–q21 (*hMSH3*), *D5S416*, *D5S477*, *D5S651*, *D5S674*, *D5S426*, *D5S395*, *D5S418*, *D5S430*, *D5S491*, *D5S398*, *D5S431*, *D5S624*, *D5S427*, *D5S668, D5S647*, *D5S629*, *D5S428* and *D5S433*; on 2q32 (*hPMS1*), D2S318 and D2S118; on 2p16 (*hMSH2/hMSH6*), D2S391 and D2S288; on 7p22 (*hPMS2*), D7S531 and D7S517. The corresponding PCR primers were provided by Research Genetics. The chromosomal assignment of these microsatellites and genes was performed by integrating genetic, radiation hybrid and STS/YAC data from several sources (Gyapay et al, 1994; Hudson et al, 1995; Gemmill et al, 1995). Thirty amplification cycles of 1 min at 94°C, 1 min at 50–60°C and 1 min at 72°C were carried out in 20 µl of 10 mM tris-HCl (pH 8.3), 50 mM potassium chloride, 1.5 mM magnesium chloride containing 0.2 µM of each primer, 50 µM dNTPs, 1 µCi of [$^{32}$P]αCTP (ICN; specific activity 3000 Ci mmol$^{-1}$), 5 ng of genomic DNA, and 0.4 U *Taq* DNA polymerase (BRL). The products were fractionated by denaturing electrophoresis in a 6% polyacrylamide gel, subsequently dried and autoradiographed. LOH was defined visually as the disappearance or significant reduction in the intensity of one allele in tumoral DNA compared with the normal DNA sample as described in Baccichet et al (1997). Only informative (heterozygous) loci were considered for LOH frequency calculations.

### Single-strand conformational polymorphism (SSCP) analysis

The typing of *hMLH1* exon 8 polymorphism by SSCP analysis using previously published oligonucleotides (Han et al, 1995) was performed as described in Zietkiewicz et al (1992).

### RESULTS

Our microsatellite analysis (Figure 1) revealed LOH in 10 out of the 22 patients in at least one of the mismatch-repair loci tested (Table 1).

### Detection of LOH on chromosome 3p21

Out of the 22 patients, ten (46%) exhibited LOH in at least one of the microsatellite markers located on chromosome 3p21. Patient 3 lost an allele at *D3S1745* and *D3S1561*, but maintained heterozygosity at the distal neighbouring locus *D3S1266*; patient 43 showed LOH at every marker distal to *D3S1611* and *D3S1612*, but retained both alleles at *D3S1298* (Figure 2). These results suggested that the shortest region of overlapping (SRO) deletions delimited by *D3S1298* and *D3S1266* included *hMLH1* at 3p21–p22 (Figure 2). In addition to the intragenic *D3S1611* marker (Papadopoulos et al, 1994), we analysed by SSCP a biallelic polymorphism in exon 8 of *hMLH1* to show hemizygous deletion in the three informative cases (not shown).

Several genes have been shown to be included in LOH regions on chromosome 3p (Figure 2). We extended the allelotyping to investigate the possible involvement of the SCLC region, which was shown to be homozygously deleted in small-cell lung cancer cell lines (Daly et al, 1993), as well as the *FHIT* and *PTPRG* genes (Figure 2). Among the ten patients with LOH at *hMLH1*, four

**Table 1** Summary of the LOH data for the analysed mismatch repair-related chromosomal regions

| Cases | DNA mismatch-repair-related region | | | | |
|---|---|---|---|---|---|
| | ***hMLH1*** **(3p21)** | ***hMSH3*** **(5q11–q13)** | ***hPMS1*** **(2q32)** | ***hMSH2 hMSH6*****(2p16)** | ***hPMS2*** **(7p22)** |
| 1 | LOH | H | H | H | H |
| 3 | LOH | H | H | NI | H |
| 5 | H | H | H | H | H |
| 7 | H | H | NI | H | NI |
| 9 | LOH | LOH | LOH | LOH | LOH |
| 11 | LOH | LOH | LOH | H | H |
| 13 | LOH | LOH | H | H | H |
| 15 | H | H | H | H | H |
| 17 | LOH | H | H | H | H |
| 19 | H | H | H | H | H |
| 21 | H | H | NI | H | H |
| 23 | H | H | H | NI | H |
| 25 | H | H | H | H | H |
| 27 | H | H | H | H | H |
| 29 | LOH | LOH | H | H | H |
| 31 | H | H | H | H | H |
| 33 | LOH | H | H | H | H |
| 35 | H | H | H | H | H |
| 37 | LOH | LOH | H | H | H |
| 39 | H | H | H | H | H |
| 41 | H | H | H | H | H |
| 43 | LOH | H | H | H | H |
| LOH frequency | 45.45% | 22.7% | 10% | 5% | 4.7% |

LOH, loss of heterozygosity, H, heterozygote, NI, non-informative.

© Cancer Research Campaign 1999

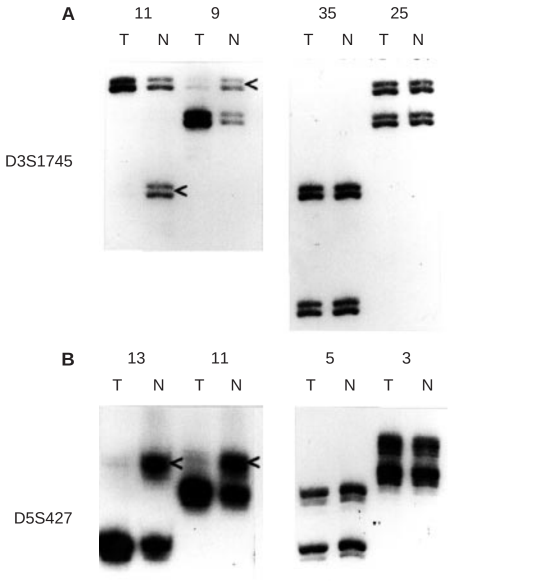

**Figure 1** Examples of LOH and heterozygosity of the 3p21 and 5q11–q13 regions. Patients 9, 11, 25 and 35 analysed with marker D3S1745 and patients 3, 5, 11 and 13 analysed with marker D5S427; arrowheads indicate the deleted alleles in tumours; (N) normal and (T) tumoral DNA

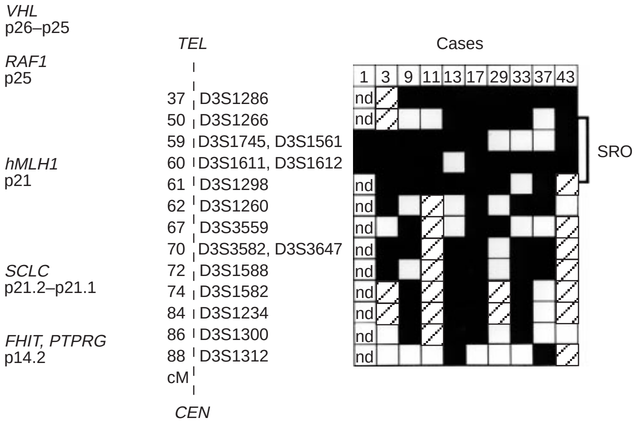

**Figure 2** LOH profiles around the 3p21 region and the shortest region of overlapping deletion (SRO). ■, LOH; ◪, no LOH; □, non-informative; nd, not done

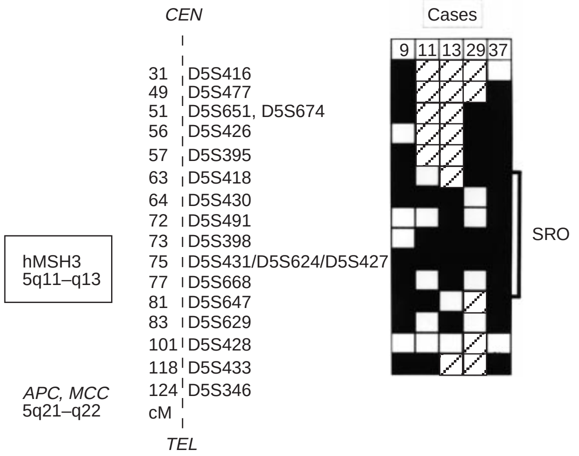

**Figure 3** LOH profiles around the 5q11–q13 region and the shortest region of overlapping deletion (SRO). ■, LOH; ◪, no LOH; □, non-informative retained heterozygosity at proximal markers (Figure 2). For instance, in patient 11, markers *D3S1298* through *D3S1286* revealed LOH, but at markers proximal to *D3S1260* both alleles were retained thus excluding the SCLC region as well as *FHIT* and *PTPRG* as deletion targets. Similarly, these loci were excluded from the SRO in patient 43 (Figure 2). The SCLC region delimited by markers *D3S1588* and *D3S1613* (Daly et al, 1993) was partly affected by LOH in patients 3 and 29. Particular attention was placed on *FHIT* because abnormal transcription of this gene was reported in 30% of breast cancer patients (Negrini et al, 1996). Markers linked to the *FHIT* locus, including *D3S1300* which maps within intron 5 of the *FHIT* gene (Man et al, 1996), were deleted in 4 out of the 11 cases informative at this locus (36%), which were all also deleted for *hMLH1* (not shown). Therefore, 3p deletions represent two groups, one with small deletions affecting the *hMLH1* locus and another with larger deletions that include the *hMLH1*, SCLC and *FHIT* loci (Figure 2).

### Detection of LOH on chromosome 5q11–q13

As shown in Figure 3, 5 out of the 22 informative cases (23%) were hemizygously deleted at one or more marker loci tightly linked to the *hMSH3* locus. All of these patients with LOH at 5q11–q13 were also deleted in the 3p21 region (Figure 2 and 3). This non-random distribution of concomitant deletions was statistically significant ($P \sim 0.02$, chi-squared test). Large deletions were seen in patients 9 and 37, whereas others exhibited restricted LOH: patient 13 with LOH at *D5S430* retained heterozygosity at every marker proximal to *D5S418*, whereas patient 29 was heterozygous at *D5S647* thus delimiting the SRO between *D5S418* and *D5S647* at 5q11–q13 (Figure 3). The tumour-suppressor genes *APC* and *MCC* both located on chromosome 5q21 were excluded from deletions involving *hMSH3* in two of the patients (13 and 29) heterozygous for markers linked to *APC* and *MCC* regions (Figure 3). Thus, *hMSH3* is a good candidate as the target of 5q11–q13 deletions.

In contrast to *hMLH1* and *hMSH3*, we found only two patients that were affected by allelic deletions at other tested loci: 1 out of 21 informative cases at *hPMS2*, 1 out of 20 at *hMSH2/hMSH6* and 2 out of 20 at *hPMS1* (Table 1), including patient 9 who displayed LOH at all investigated loci. The low rate of allelic losses (5–10%) affecting the chromosomes 2p16, 2q31–q33 and 7p15–pter may reflect the baseline frequency of LOH in breast cancer (Chen et al, 1992).

### Microsatellite instability

None of the 22 tumours displayed the RER phenotype as judged by the absence of instability at the 42 microsatellites tested (a total of 572 independent comparisons). Although our markers were already shown as sensitive to detecting RER in sporadic colorectal cancers (Benachenhou et al, 1998*a*), we additionally examined two markers *GGAA4D07* and *GGAA2E02* recently reported as unstable in 30% (11 out of 37) and 41% (15 out of 37) of breast cancer patients respectively (Paulson et al, 1996). These two markers did not reveal any instability in the 22 tumours examined here (data not shown). Our findings were thus consistent with studies of two large cohorts of breast cancer patients that failed to detect significant levels of microsatellite instability (Lothe et al, 1993; Wooster et al, 1994).

 © Cancer Research Campaign 1999

### DISCUSSION

The activation of oncogenes, the loss or inactivation of repressor genes and impaired mismatch-repair function are known to be involved in the development of solid tumours. Defects in DNA mismatch-repair genes lead to replication errors revealed as instability in microsatellite markers (Leach et al, 1993; Bronner et al, 1994; Papadopoulos et al, 1994). A proposal that deficient DNA repair was a predisposing factor in sporadic breast cancer (Helzlsouer et al, 1996; Parshad et al, 1996) was promoted by reports of microsatellite instability in breast tumours (Yee et al, 1994; Karnik et al, 1995; Paulson et al, 1996). By allelotyping the mismatch-repair genes *hMLH1*, *hMSH2*, *hMSH3*, *hMSH6*, *hPMS1* and *hPMS2*, we have shown that 46% and 23% of the breast tumours tested were affected by allelic losses at *hMLH1* and *hMSH3* respectively. Because none of the tumour tissues were microdissected, these figures should be considered conservative as some allelic losses could have been masked by contaminating genetic material of normal cells. Other alterations such as small deletions, point mutations, gene rearrangements, or DNA methylation, if they also contribute to inactivation of these loci, could escape detection by our approach. Further studies are required to explore these possibilities.

Interstitial deletion of chromosome 3p is one of the most common genetic rearrangements observed in tumour cells (Pandis et al, 1993). The region 3p14–p23 has been shown to be deleted in small-cell lung carcinomas (Petersen et al, 1997), non-small-cell lung carcinomas (Benachenhou et al, 1998*b*) renal cell carcinomas (Foster et al, 1994) and uterine cervical carcinomas (Kohno et al, 1993). In breast cancer, LOH ranging from 30% to 47% were observed at two separate regions, 3p13–p14 and 3p21–p25 (Chen et al, 1994) or 3p14.3–p21.1 and 3p24.3–p25.1 (Matsumoto et al, 1997), suggesting the involvement of several tumour-suppressor genes. *PTPRG*, a protein-tyrosine phosphatase gene, and *FHIT* that encodes the human diadenosine triphosphate hydrolase (Barnes et al, 1996), both localized within the 3p13–p14 region, are potential candidates as targets of deletions in primary breast tumours (LaForgia et al, 1991; Negrini et al, 1996; Ohta et al, 1996). Furthermore, a 3p21.3 region (SCLC) was shown to be homozygously deleted in SCLC cell lines (Daly et al, 1993). Our allelotyping data narrow down the critically deleted region on chromosome 3p21 (Figure 2) to an interval delimited by markers *D3S1298* and *D3S1266*, thus excluding SCLC, *FHIT* and *PTPRG* from the SRO (Figure 2). In another study, we showed that the smallest region of overlapping deletions in non-small-cell lung cancer patients was refined to a 1-cM interval between markers *D3S1561* and *D3S1612* (Benachenhou et al, 1998*b*). The only known candidate which remains in the deleted region is thus *hMLH1*. Do hemizygous deletions of this gene promote cancer progression, or is *hMLH1* only in linkage to a yet unidentified tumour-suppressor gene needs to be investigated further?

Deletions of chromosome 5q were previously reported in several tumour types, including colorectal cancers (Solomon et al, 1987; Vogelstein et al, 1988; Ashton-Rickart et al, 1991), lung cancers (Ashton-Rickart et al, 1991; D'Amico et al, 1992; Benachenhou et al, 1998*b*), and oesophageal cancers (Boynton et al, 1992). At least two known tumour suppressors, *APC* and *MCC*, are localized within this region. Reports of 5q21 deletions in 18–29% of sporadic breast cancers suggests the involvement of *APC* and *MCC* (Thompson et al, 1993; Medeiros et al, 1994). Although some deletions on 5q overlap with the *APC*/*MCC* region, our results exclude these loci from the SRO in 40% of the 5q deleted patients. Delimited by *D5S418* and *D5S647*, this SRO is 43.5 cM away from *APC* and *MCC* (Figure 3). Allelic losses at 5q13.1–q21 in 33% of ovarian cancers (Tavassoli et al, 1996) as well as in 42% of NSCLC (Benachenhou et al, 1998*b*) also occurred outside the *APC*-containing region. Thus, genes other than *APC*/*MCC* have to be considered as targets of 5q11–q13 deletions. *hMSH3* is a good candidate as the target of 5q11–q13 deletions, although the existence of a yet unidentified adjacent gene cannot be ruled out.

None of the tumors analysed in this study displayed microsatellite instability, a hallmark of a deficiency in the replication errors correction. Expecting a 30% incidence of instability as reported by Paulson et al (1996), the probability of not detecting a single unstable tumour in our sample of 22 was as low as 0.0014 ($Po = e^{-0.3 \times 22}$). The absence of RER is, however, consistent with earlier reports indicating a virtual absence of microsatellite instability in breast tumour cells (Han et al, 1993; Lothe et al, 1993; Wooster et al, 1994). Moreover, considering four studies (Han et al, 1993; Lothe et al, 1993; Wooster et al, 1994; this study) in which no evidence of RER was obtained (i.e. no more than a single instability per sample), we estimated the average rate of somatic microsatellite mutations at $5 \times 10^{-3}$ (12 out of 2556), a frequency similar to the one from T-lymphocytes ($3 \times 10^{-3}$) (Hackman et al, 1995). It is difficult to explain the variability in the reported prevalence of RER+ breast tumours ranging from 0% to 30%. Because the random sampling effect was rather unlikely, this discrepancy could be related to sample stratification, to criteria used to define RER+ tumours (Dietmaier et al, 1997) and the nature of the markers used to reveal this phenotype (Arzimanoglou et al, 1998), or requires other explanations.

We are, thus, left with patients associated with hemizygous deletions at two mismatch-repair loci and no RER. If the non-deleted *hMLH1* and *hMSH3* alleles are still active, this could explain the absence of a RER phenotype. At this point, it is difficult to decide whether hemizygous deletions of *hMLH1* and *hMSH3* genes promote breast tumorigenesis, or whether LOH at these loci only indicates linkage with as yet unknown tumour-suppressor loci. However, concomitant deletions of *hMSH3* and *hMLH1* in a number of patients raise unanswered questions about their relationship. *hMSH2* and *hMSH6* or *hMSH3* proteins bind the mismatch as heterodimers called, respectively, $hMutS\alpha$ and $hMutS\beta$ (Drummond et al, 1995; Palombo et al, 1996), which are then recognized by the heterodimer $hMutL\alpha$ composed of *hMLH1*/*hPMS2*. Therefore, a gene dosage effect affecting the stoichiometry and the activity of the heteromolecular mismatch-repair complex may be sufficient to promote cancer by impairing functions other than the correction of replication errors. Mismatch-repair proteins are involved in a variety of vital cellular processes, including the homologous recombination (Jones et al, 1987; deWind et al, 1995), the mediation of the G2 checkpoint (Hawn et al, 1995), transcription-coupled nucleotide excision repair (Mellon et al, 1996) and in the recognition of DNA damage and/or in the signalling pathway contributing to the generation of apoptotic cells (Kat et al, 1993; Mu et al, 1997). Interestingly, the influence of environmental factors on the genome stability in cells defective in nucleotide excision repair and mismatch-repair could be substantial. In this regard, it has been recently demonstrated that homozygous as well as heterozygous *hMSH2* mutant mammalian cells have a propensity to accumulate potentially mutagenic oxidative DNA damage (DeWeese et al, 1997) that may promote the

© Cancer Research Campaign 1999

development of cancer through defects in genes sensitive to exogenous factors (Mellon et al, 1996; DeWeese et al, 1997). In addition, a heterozygous mutation in *hMLH1* in a human-derived cancer cell line was shown to significantly reduce transcription-coupled repair involved in selective removal of DNA damage from the transcribed strands of active genes (Mellon et al, 1996*b*), pointing to the possibility that allelic deletion of *hMLH1* and/or *hMSH3* could have the same effect. We propose that a subtle defect in the repair of DNA damage, which is less likely to be lethal to the carrying cells, could have an even more profound impact on tumorigenesis, thus placing individuals at increased cancer risk.

In conclusion, our allelotyping analysis of sporadic breast carcinomas demonstrated that two DNA mismatch-repair loci, *hMLH1* and *hMSH3*, are frequently affected by LOH at chromosomal regions 3p21 and 5q11–q13 respectively. We suggest that *hMLH1* and *hMSH3* deletion could promote cancer progression through a dosage effect affecting cellular functions other than replication errors correction. Whether or not *hMLH1* and *hMSH3* are real targets of the deletions is still under investigation, but the identification of genes with suppressor activity for malignancy at 3p21 and 5q11–q13 is extremely important considering the frequency of LOH at these regions in several major forms of cancer.

### ACKNOWLEDGEMENTS

NB has a fellowship from the Medical Research Council of Canada. SG has a studentship of the Fonds pour la Formation de Chercheurs et l'Aide à la Recherche. DS is a scholar of the Fonds de la recherche en Santé du Québec. This work was supported by the Fondation Charles Bruneau.

### REFERENCES

Arzimanoglou II, Gilbert F and Barber HRK (1998) Microsatellite instability in human solid tumors. *Cancer* **82**: 1808–1820

Ashton-Rickart PG, Wyllie AH, Bird CC, Dunlop MG, Steel CM, Morris RG, Piris J, Romanowski P, Wood R, White R, Nakamura Y (1991) MCC, a candidate familial polyposis gene in 5q21 shows frequent allele loss in colorectal and lung cancer. *Oncogene* **6**: 1881–1886

Baccichet A, Qualman SA and Sinnett D (1997) Allelic loss in childhood acute lymphoblastic leukemia. *Leukemia Res* **21**: 817–823

Barnes LD, Garrison PN, Siprashvili Z, Guranowski A, Robinson AK, Ingram SW, Croce CM, Ohta M and Huebner K (1996) Fhit, a putative tumor suppressor in humans, is a dinucleoside 5′,5′′′-P1,P3 triphosphate hydrolase. *Biochemistry* **35**: 11529–11535

Benachenhou N, Guiral S, Gorska-Flipot I, Michalski R, Labuda D and Sinnett D (1998*a*) Allelic losses and DNA methylation at DNA mismatch-repair loci in sporadic colorectal cancer. *Carcinogenesis* (in press)

Benachenhou N, Guiral S, Gorska-Flipot I, Labuda D and Sinnett D (1998*b*) High resolution deletion mapping reveals frequent allelic losses at the DNA mismatch-repair loci in *hMLH1* and *hMSH3* in non-small cell lung cancer. *Int J Cancer* **77**: 173–180

Bièche I and Lidereau R (1995) Genetic alterations in breast cancer. *Genes Chromosomes Cancer* **14**: 227–251

Boynton RF, Blount PL, Yin J, Brown VL, Huang Y, Tong Y, McDaniel T, Newkirk C and Resau JH (1992) Loss of heterozygosity involving the APC and MCC genetic loci occurs in the majority of human esophageal cancers. *Proc Natl Acad Sci USA* **89**: 3385–3388

Bronner CE, Baker SM, Morrison PT, Warren G, Smith LG, Lescoe MK, Kane M, Earabino C, Lipford J, Lindblom A, Tannergard P, Bollag RJ, Godwin AR, Ward DC, Nordenskjold M, Fishel R, Kolodner R and Liskay RM (1994) Mutation in the DNA mismatch repair gene homologue hMLH1 is associated with hereditary non-polyposis colon cancer. *Nature* **368**: 258–261

Chen LC, Kurisu W, Ljung BM, Goldman ES, Moore Jr D and Smith HS (1992) Heterogeneity for allelic loss in human breast cancer. *J Natl Cancer Inst* **84**: 506–510

Chen LC, Matsumura K, Deng G, Kurisu W, Ljung BM, Lerman MI, Waldman FM and Smith HS (1994) Deletion of two separate regions on chromosome 3p in breast cancers. *Cancer Res* **54**: 3021–3024

Cornelisse CJ, Kuipers-Dijkshoorn N, van Vliet M, Hermans J and Devilee P (1992) Fractional allelic imbalance in human breast cancer increases with tetraploidization and chromosome loss. *Int J Cancer* **50**: 544–548

Daly MC, Xiang RH, Buchhagen D, Hensel CH, Garcia DK, Killary AM, Minna J and Naylor SL (1993) A homozygous deletion on chromosome 3 in a small cell lung cancer cell line correlates with a region of tumor suppressor activity. *Oncogene* **8**: 1721–1729

D'amico D, Carbone DP, Johnson BE, Meltzer J and Minna JD (1992) Polymorphic sites within the MCC and APC loci reveal very frequent loss of heterozygosity in human small cell lung cancer. *Cancer Res* **52**: 1996–1999

DeWeese TL, Bucci JM, Larrier NA, Cutler RG, te Riele H and Nelson WG (1997) Tolerance of oxidative DNA damage results from disruption of *Msh2* alleles: implications for carcinogenesis. In *Proceedings of the 88th Annual Meeting of the American Association for Cancer Research*, Vol. 38, AACR (eds). CADMUS Journal Services: Lithicum MD, USA

deWind N, Dekker M, Berns A, Radman M and te Riele H (1995) Inactivation of the mouse Msh2 gene results in mismatch repair deficiency, methylation tolerance, hyperrecombination, and predisposition to cancer. *Cell* **82**: 321–330

Dietmaier W, Wallinger S, Bocker T, Kullmann F, Fishel R and Rüschoff J (1997) Diagnostic microsatellite instability: definition and correlation with mismatch repair protein expression. *Cancer Res* **57**: 4749–4756

Drummond JT, Li GM, Longley MJ and Modrich P (1995) Isolation of an hMSH2-p160 heterodimer that restores DNA mismatch repair to tumor cells. *Science* **268**: 1909–1912

Fishel R, Lescoe MK, Rao MRS, Copeland NG, Jenkins NA, Garber J, Kane M and Kolodner R (1993) The human mutator gene homolog MSH2 and its association with hereditary nonpolyposis colon cancer. *Cell* **75**: 1027–1038

Foster K, Crossey PA, Cairns P, Hetherington JW, Richards FM, Jones MH, Bentley E, Affara NA, Ferguson-Smith MA and Maher ER (1994) Molecular genetic investigation of sporadic renal cell carcinoma: analysis of allele loss on chromosomes 3p, 5q, 11p, 17 and 22. *Br J Cancer* **69**: 230–234

Gemmill RM, Chumakov I, Scott P, Waggoner B, Rigault P, Cypser J, Chen Q, Weissenbach J, Gardiner K, Wang H, Pekarsky Y, Le Gall I, Le Paslier D, Guillou S, Li E, Robinson L, Hahner L, Todd S, Cohen D and Drabkin HA (1995) A second-generation YAC contig map of human chromosome 3. *Nature* **377** (suppl.): 299–319

Gyapay G, Morissette J, Vignal A, Dib C, Fizames C, Millasseau P, Marc S, Bernardi G, Lathrop M and Weissenbach J (1994) The 1993–94 Genethon human genetic linkage map. *Nature Genet* **7**: 246–339

Hackman P, Gabbani G, Österholm A-M, Hellgren D and Lambert B (1995) Spontaneous length variation in microsatellite DNA from human T-cell clones. *Genes Chromosomes Cancer* **14**: 215–219

Han HJ, Yanagisawa A, Kato Y, Park JG and Nakamura Y (1993) Genetic instability in pancreatic cancer and poorly differentiated type of gastric cancer. *Cancer Res* **53**: 5087–5089

Han HJ, Maruyama M, Baba S, Park JG and Nakamura Y (1995) Genomic structure of human mismatch repair gene, *hMLH1*, and its mutation analysis in patients with hereditary non-polyposis colorectal cancer (HNPCC). *Hum Mol Genet* **4**: 237–242

Hawn MT, Umar A, Carethers JM, Marra G, Kunkel TA, Boland CR and Koi M (1995) Evidence for a connection between the mismatch repair system and the G2 cell cycle checkpoint. *Cancer Res* **55**: 3721–3725

Helzlsouer KJ, Harris EL, Parshad R, Perry HR, Price FM and Sanford KK (1996) DNA repair proficiency: potential susceptibility factor for breast cancer. *J Natl Cancer Inst* **88**: 754–755

Hemminki A, Peltomaki P, Mecklin JP, Jarvinen H, Salovaara R, Nystrom-Lahti M, de la Chapelle A and Aaltonen LA (1994) Loss of the wild-type *MLH1* gene is a feature of hereditary nonpolyposis colorectal cancer. *Nature Genet* **8**: 405–410

Hudson TJ, Stein LD, Gerety SS, Ma J, Castle AB, Silva J, Slonim DK, Baptista R, Kruglyak L, Xu S-H, Hu X, Colbert AME, Rosenberg C, Reeve-Daly MP, Rozen S, Hui L, Wu X, Vestergaard C, Wilson KM, Bae JS, Maitra S, Ganiatsas S, Evans CA, DeAngelis MM, Ingalls KA, Nahf RW, Horton Jr, LT, Anderson MO, Collymore AJ, Ye W, Kouyoumjian V, Zemsteva IS, Tam J, Devine R, Courtney DF, Renaud MT, Nguyen H, O'Connor TJ, Fizames C, Fauré S, Gyapay G, Dib C, Morissette J, Orlin JB, Birren BW, Goodman N, Weissenbach J, Hawkins TL, Foote S, Page DC and Lander ES (1995) An STS-based map of the human genome. *Science* **270**: 1945–1954

Jones M, Wagner R and Radman M (1987) Mismatch repair and recombination in *E. coli*. Cell **50**: 621–626

 © Cancer Research Campaign 1999

Karnik P, Plummer S, Casey G, Myles J, Tubbs R, Crowe J and Williams BR (1995) Microsatellite instability at a single locus (*D11S988*) on chromosome 11p15.5 as a late event in mammary tumorigenesis. *Hum Mol Genet* **4**: 1889–1894

Kat A, Thilly WG, Fang WH, Longley MJ, Li GM and Modrich P (1993) An alkylation-tolerant, mutator human cell line is deficient in strand-specific mismatch repair. *Proc Natl Acad Sci USA* **90**: 6424–6428

Knudson Jr AG (1971) Mutation and cancer: statistical study of retinoblastoma. *Proc Natl Acad Med* **68**: 820–823

Kohno T, Takayama H, Hamaguchi M, Takano H, Yamaguchi N, Tsuda H, Hirohashi S, Vissing H, Shimizu M and Oshimura M (1993) Deletion mapping of chromosome 3p in human uterine cervical cancer. *Oncogene* **8**: 1825–1832

LaForgia SK, Morse B, Levy J, Barnea G, Li F, Cannizarro LA, Nowell PC, Glick J, Boghosian-Sell L, Weston A, Harris CC, Drabkin H, Patterson D, Croce CM, Schlessinger J and Huebner K (1991) Receptor protein-tyrosine phosphatase is a candidate tumor suppressor gene at human chromosome region 3p21. *Proc Natl Acad Sci USA* **88**: 5036–5040

Leach FS, Nicolaides NC, Papadopoulos N, Liu B, Jen J, Parsons R, Peltomaki P, Sistonen P, Aaltonen LA, Nystrom-Lahti M, Guan XY, Zhang J, Meltzer PS, Yu JW, Kao FT, Chen DJ, Cerosaletti KM, Fournier REK, Todd S, Lewis T, Leach RJ, Naylor SL, Weissenbach J, Mecklin JP, Järvinen H, Petersen GM, Hamilton SR, Green J, Jass J, Watson P, Lynch TT, Trent JM, de la Chapelle A, Kinzler KW and Vogelstein B (1993) Mutations of a mutS homolog in hereditary nonpolyposis colorectal cancer. *Cell* **75**: 1215–1225

Liu B, Nicolaides NC, Markowitz S, Willson JK, Parsons RE, Jen J, Papadopolous N, Peltomaki P, de la Chapelle A, Hamilton SR, Kinzler KW and Vogelstein B (1995) Mismatch repair gene defects in sporadic colorectal cancers with microsatellite instability. *Nature Genet* **9**: 48–55

Lothe RA, Peltomaki P, Meling GI, Aaltonen LA, Nystrom-Lahti M, Pylkkanen L, Heimdal K, Andersen TI, Moller P, Rognum TO, Fossa SD, Haldorsen T, Langmark F, Brogger A, de la Chapelle A and Borresen AL (1993) Genomic instability in colorectal cancer: relationship to clinicopathological variables and family history. *Cancer Res* **53**: 5849–5852

Man S, Ellis IO, Sibbering M, Blamey RW and Brook JD (1996) High levels of allele loss at the *FHIT* and *ATM* genes in non-comedo ductal carcinoma in situ and grade I tubular invasive breast cancers. *Cancer Res* **56**: 5484–5489

Matsumoto S, Kasumi F, Sakamoto G, Onda M, Nakamura Y and Emi M (1997) Detailed deletion mapping of chromosome arm 3p in breast cancers: a 2-cM region on 3p14.3–21.1 and a 5-cM region on 3p24.3–25.1 commonly deleted in tumors. *Genes Chromosomes Cancer* **20**: 268–274

Medeiros AC, Nagai MA, Neto MM and Brentani RR (1994) Loss of heterozygosity affecting the APC and MCC genetic loci in patients with primary breast carcinomas. *Cancer Epidemiol Biomarkers Prev* **3**: 331–333

Mellon I, Rajpal DK, Koi M, Boland CR and Champe N (1996) Transcription-coupled deficiency and mutations in mismatch repair genes. *Science* **272**: 557–560

Mu D, Turson M, Duckett DR, Drummond JT, Modrich P and Sanxcar A (1997) Recognition and repair of compound DNA lesions (base damage and mismatch) by human mismatch repair and excision repair systems. *Mol Cell Biol* **17**: 760–769

Negrini M, Monaco C, Vorechovsky I, Ohta M, Druck T, Baffa R, Huebner K and Croce CM (1996) The *FHIT* gene at 3p14.2 is abnormal in breast carcinomas. *Cancer Res* **56**: 3173–3179

Newman B, Austin MA, Lee M and King MC (1988) Inheritance of human breast cancer: evidence for autosomal dominant transmission in high risk families. *Proc Natl Acad Sci USA* **85**: 3044–3048

Nicolaides NC, Papadopoulos N, Liu B, Wei YF, Carter KC, Ruben SM, Rosen CA, Haseltine WA, Fleischmann RD, Fraser CM, Adams MD, Venter JC, Dunlop MG, Hamilton SR, Peterson GM, de la Chapelle A, Vogelstein B and Kinzler KW (1994) Mutations of two PMS homologues in hereditary nonpolyposis colon cancer. *Nature* **371**: 75–80

Ohta M, Inoue H, Cotticelli MG, Kastury K, Baffa R, Palazzo J, Siprashvili Z, Mori M, McCue P, Druck T, Croce CM and Huebner K (1996) The human *FHIT* gene, spanning the chromosome 3p14.2 fragile site and renal carcinoma associated translocation breakpoint, is abnormal in digestive tract cancers. *Cell* **84**: 587–597

Palombo F, Iaccarino I, Nakajima E, Ikejima M, Shimada T and Jiricny J (1996) hMutSbeta, a heterodimer of hMSH2 and hMSH3, binds to insertion/deletion loops in DNA. *Curr Biol* **6**: 1181–1184

Pandis N, Jin Y, Limon J, Bardi G, Idvall I, Mandahl N, Mitelman F and Heim S (1993) Interstitial deletion of the short arm of chromosome 3 as a primary chromosome abnormality in carcinomas of the breast. *Genes Chromosomes Cancer* **6**: 151–155

Papadopoulos N, Nicolaides NC, Wei YF, Ruben SM, Carter KC, Rosen CA, Haseltine WA, Fleischmann RD, Fraser CM, Adams MD, Venter, JC, Hamilton SR, Petersen GM, Watson P, Lynch HT, Peltomäki P, Mecklin JP, de la Chapelle A, Kinzler KW and Vogelstein B (1994) Mutation of a mutL homolog in hereditary colon cancer. *Science* **263**: 1625–1629

Papadopoulos N, Nicolaides NC, Liu B, Parsons R, Lengauer C, Palombo F, D'Arrigo A, Markowitz S, Willson JK, Kinzler KW, Jiricny J and Vogelstein B (1995) Mutations of GTBP in genetically unstable cells. *Science* **268**: 1915–1917

Parshad R, Price FM, Bohr VA, Cowans KH, Zujewski JA and Sanford KK (1996) Deficient DNA repair capacity, a predisposing factor in breast cancer. *Br J Cancer* **74**: 1–5

Paulson TJ, Wrught FA, Parker BA, Russack V and Walh GM (1996) Microsatellite instability correlates with reduced survival and poor disease prognosis in breast cancer. *Cancer Res* **56**: 4021–4026

Petersen I, Langreck H, Wolf G, Schwendel A, Psille R, Vogt P, Reichel MB, Ried T and Dietel M (1997) Small-cell lung cancer is characterized by a high incidence of deletions on chromosomes 3p, 4q, 5q, 10q, 13q and 17p. *Br J Cancer* **75**: 79–86

Risinger JI, Umar A, Boyd J, Berchuck A, Kunkel A and Barrett JC (1996) Mutation in *MSH3* in endometrial cancer and evidence for its functional role in repair. *Nature Genet* **14**: 102–105

Sato T, Tanigami A, Yamakawa K, Akiyama F, Kasumi F, Sakamoto G and Nakamura Y (1990) Allelotype of breast cancer: cumulative allele losses promote tumor progression in primary breast cancer. *Cancer Res* **50**: 7184–7189

Solomon E, Voss R, Hall V, Bodmer WF, Jass JR, Jeffreys AJ, Lucibello FC, Patel I and Rider SH (1987) Chromosome 5 allele loss in human colorectal carcinomas. *Nature* **328**: 616–619

Speicher MR (1995) Microsatellite instability in human cancer. *Oncol Res* **7**: 267–275

Tavassoli M, Steingrimsdottir H, Pierce E, Jiang X, Alagoz M, Farzaneh F and Campbell IG (1996) Loss of heterozygosity on chromosome 5q in ovarian cancer is frequently accompanied by TP53 mutation and identifies a tumor suppressor gene locus at 5q13.1–21. *Br J Cancer* **74**: 115–119

Thompson AM, Morris RG, Wallace M, Wyllie M., Steel CM and Carter DC (1993) Allele loss from 5q21 (APC/MCC) and 18q21 (DCC) and DCC mRNA expression in breast cancer. *Br J Cancer* **68**: 64–68

Tomlinson IP, Ilyas M and Bodmer WF (1996) Allele loss occurs frequently at *hMLH1*, but rarely at *hMSH2*, in sporadic colorectal cancers with microsatellite instability. *Br J Cancer* **74**: 1514–1517

Vogelstein B, Fearon ER, Hamilton SR, Kern SE, Preisinger AC, Leppert M, Nakamura Y, White R, Smits AM and Bos JL (1988) Genetic alterations during colorectal-tumor development. *N Engl J Med* **319**: 525–532

Wooster R, Cleton-Jansen AM, Collins N, Mangion J, Cornelis RS, Cooper CS, Gusterson BA, Ponder BA, von Deimling A, Wiestler OD, Cornelisse CJ, Devilee P and Stratton MR (1994) Instability of short tandem repeats (microsatellites) in human cancers. *Nature Genet* **6**: 152–156

Yee CJ, Roodi N, Verrier CS and Parl FF (1994) Microsatellite instability and loss of heterozygosity in breast cancer. *Cancer Res* **54**: 1641–1644

Zietkiewicz E, Sinnett D, Richer C, Mitchell G, Vanasse M and Labuda D (1992) Single-strand conformational polymorphism (SSCP): detection of useful polymorphisms at the dystrophin locus. *Hum Genet* **89**: 453–456

© Cancer Research Campaign 1999
